# Experts’ opinion for improving global adolescent vaccination rates: a call to action

**DOI:** 10.1007/s00431-019-03511-8

**Published:** 2020-02-18

**Authors:** Chiara Azzari, Javier Diez-Domingo, Evelyn Eisenstein, Saul N. Faust, Andreas Konstantopoulos, Gary S. Marshall, Fernanda Rodrigues, Tino F. Schwarz, Catherine Weil-Olivier

**Affiliations:** 1 Department of Health Sciences, University of Florence and Meyer Children’s Hospital, viale Pieraccini 24, 50139 Florence, Italy; 2 FISABIO, Avda de Catalunya, 21, 4620 Valencia, Spain; 3 University of the State of Rio de Janeiro, - UERJ Bloco C - 9º andar, R. São Francisco Xavier, 524 - Maracanã, 20550-900 Rio de Janeiro, Brazil; 4 National Institute of Health Research Clinical Research Facility, University of Southampton and University Hospital NHS Foundation Trust, Southampton Centre for Biomedical Research, C Level West Wing, Mailpoint 218, Southampton General Hospital, Tremona Road, SO16 6YD, Southampton, UK; 5 National and Kapodistrian, University of Athens, Athens, 157 72 Greece; 6 Department of Pediatrics, University of Louisville School of Medicine, 571 S. Floyd St., Suite 321, Louisville, KY 40202 USA; 7 Hospital Pediátrico – Centro Hospitalar e Universitário de Coimbra, Praceta Prof. Mota Pinto, 3000-075 Coimbra, Portugal; 8 Institute of Laboratory Medicine and Vaccination Centre, Klinikum Wuerzburg Mitte, Standort Juliusspital, Juliuspromenade 19, 97070 Wuerzburg, Germany; 9 University of Paris 7 Denis Diderot, 28 rue Parmentier, 92200 Neuilly sur Seine, France

**Keywords:** Immunization, Vaccination, Preventive healthcare, Adolescence, Vaccine-preventable diseases

## Abstract

Worldwide, lifestyle and resource disparities among adolescents contribute to unmet health needs, which have crucial present and future public health implications for both adolescents and broader communities. Risk of infection among adolescents is amplified by biological, behavioral, and environmental factors; however, infectious diseases to which adolescents are susceptible are often preventable with vaccines. Beyond these concerns, there is a lack of knowledge regarding adolescent vaccination and disease risk among parents and adolescents, which can contribute to low vaccine uptake. Promising efforts have been made to improve adolescent vaccination by programs with motivational drivers and comprehensive communication with the public. In May 2017, a multidisciplinary group of experts met in Amsterdam, Netherlands, to discuss adolescent vaccine uptake, as part of an educational initiative called the Advancing Adolescent Health Spring Forum. This article presents consensus opinions resulting from the meeting, which pertain to the burden of vaccine-preventable diseases among adolescents, reasons for low vaccine uptake, and common characteristics of successful strategies for improving adolescent vaccination.

*Conclusion*: There is an urgent “call to action,” particularly targeting healthcare providers and public health authorities, for the prioritization of adolescent vaccination as a necessary element of preventive healthcare in this age group.

**Table:** 

**What is Known:** *• Despite increased risk of certain infectious diseases, adolescent vaccination uptake remains low.*	
**What is New:** *• Barriers to adolescent vaccine uptake include lack of information regarding vaccines and disease risk, health system inadequacies, and insufficient healthcare follow-up.* *• Successful efforts to improve adolescent vaccine uptake need cohesive leadership and involvement of multiple stakeholders, as well as youth-friendly messaging; healthcare providers and policymakers should prioritize adolescent vaccination and implement proven program strategies to improve adolescent health worldwide.*

**What is Known:**

*• Despite increased risk of certain infectious diseases, adolescent vaccination uptake remains low.*

**What is New:**

*• Barriers to adolescent vaccine uptake include lack of information regarding vaccines and disease risk, health system inadequacies, and insufficient healthcare follow-up.*

*• Successful efforts to improve adolescent vaccine uptake need cohesive leadership and involvement of multiple stakeholders, as well as youth-friendly messaging; healthcare providers and policymakers should prioritize adolescent vaccination and implement proven program strategies to improve adolescent health worldwide.*

## Introduction

Adolescents, defined by the World Health Organization (WHO) as individuals between 10 and 19 years of age, account for > 15% of the world’s population [1]. Although most adolescents are healthy, premature death is not uncommon, and illness constitutes a substantial public health burden [1]. An estimated 1.2 million adolescents worldwide died in 2015 (> 3000 per day), many from preventable or treatable causes; globally, adolescents account for 6% of disease and injury burden [1, 2]. Moreover, many risk factors for adult disease begin or are consolidated during adolescence [2]. Age-related disparities among adolescents (e.g., unstable living arrangements and reduced healthcare access compared with other age groups) contribute to unmet health needs [2, 3]. Adolescent health is therefore an important global priority that concerns the lives of individuals, the adults they will become, and the prosperity and sustainability of communities [1, 2]. Accordingly, the WHO recommends preventive strategies to address healthcare concerns and improve the well-being of this age group [2].

Infectious diseases to which adolescents are prone, including ones with potentially devastating consequences [1], can sometimes be prevented by vaccination [2, 4]. The risk of infectious disease in adolescents is amplified by biological, behavioral, and environmental factors common in this age group (see next section) [5–8], rendering adolescents an important target group for vaccination programs (Box 1). Such programs must address not only insufficient vaccination coverage rates against childhood diseases and waning immunity from early childhood vaccine series but also primary vaccination against diseases uniquely affecting adolescents. Adolescent vaccination is critically important for public health, potentially contributing to community immunity (or “herd immunity”) by limiting circulation and transmission of certain infectious agents (e.g., *Neisseria meningitidis*) in the general population [9]. Finally, adolescent vaccination programs provide an important auxiliary benefit of encouraging teenager engagement in their own preventive healthcare throughout life [10].

**Table:** Box Adolescents and vaccination against vaccine-preventable diseases

Contributors to increased risk of vaccine-preventable diseases among adolescents • Social behaviors involving close contact • Missed childhood vaccinations • Waning immunity from childhood vaccinations Reasons for low vaccine uptake among adolescents • Lack of knowledge and poor communication among providers, parents, and adolescents • Structural barriers in the health system • Lack of clarity regarding ownership of vaccination • Lack of primary healthcare visits • Missed opportunities for vaccination Successful strategies for improving adolescent vaccination rates • Political will • Involvement of multiple stakeholders • Use of motivational drivers • Communication of benefits of vaccination to the general public • Use of nuanced messages by age group Presentation of information in an evidence-based and youth-friendly way	

Despite all of this, vaccine uptake among adolescents remains low compared with infant programs [11–13]. For example, in 2018, the US Centers for Disease Control and Prevention reported coverage rates of 50.8% for ≥ 2 doses of the quadrivalent meningococcal (MenACWY) vaccine and 51.1% for the human papillomavirus (HPV) vaccination series among adolescents aged 13 to 17 years [13]; for comparison, the 2017 coverage rate for ≥ 3 doses of diphtheria, tetanus, and acellular pertussis (DTaP) vaccine in children aged 19 to 35 months was 94.0% [12].

The Advancing Adolescent Health Forum, an educational initiative sponsored by Pfizer Inc, comprising a multidisciplinary panel of experts from around the globe, was created to further understand the barriers to vaccine uptake among adolescents and to develop corresponding strategies for healthcare providers (HCPs). This article presents the discussions and opinions resulting from the May 2017 forum in Amsterdam, Netherlands. It serves as a “call to action” to global HCPs and public health administrators to prioritize adolescent vaccination as a central tenet of preventive healthcare for this age group.

## Vaccine-preventable diseases and current vaccination coverage among adolescents

Adolescents are at increased risk for some vaccine-preventable diseases because of a variety of age-specific behavioral, environmental, and biological factors. For example, most of the recent outbreaks of invasive meningococcal disease in the USA have occurred on college campuses [14], where adolescents and young adults live in close quarters and socialize frequently. Common social behaviors for this age group, including frequent kissing or attending social clubs and nightclubs, also increase the risk of pathogen transmission [5–8]. Another example of increased risk of meningococcal disease from close contact is a recent outbreak in Europe that was linked to the 2015 World Scout Jamboree in Japan, which was attended by over 33,000 teenage scouts from 162 countries [8]. Additionally, although smoking rates have decreased worldwide, including in the UK and the USA [15], smoking and passive smoking are also associated with meningococcal disease [16]. Despite the increased risks among adolescents, coverage rate for ≥ 2 doses of MenACWY was only 50.8% in the USA in 2018, only a modest increase from the previous year (39.1%) [13, 17].

Another classic example of behavior-associated infection risk is HPV. Because HPV is largely transmitted through sexual contact, recommendations are designed to target adolescents before initiation of sexual activity [4]. Regardless of these recommendations, HPV vaccination coverage of at least 1 dose among adolescents is only 68.1% in the USA as of 2018 [13]. In Europe, national vaccination coverage rates range from 85.9% in some parts (the UK) to as low as 14.1% (Bulgaria), despite inclusion of the vaccine in their immunization programs [18].

Missed childhood vaccinations increase the risk of disease in adolescence, with potentially severe consequences. A 95% vaccine coverage rate with 2 doses during childhood vaccination would ideally render all adolescents immune to measles [19]. However, childhood vaccinations are often missed in clinical practice, with consequences clearly demonstrated by an ongoing measles outbreak in Europe that caused 43 deaths from January 1, 2016, to mid-September 2017 [20]. From July 2018 to June 2019, among > 9500 cases occurring across Europe with known vaccination status, 70% of cases were unvaccinated; 18% received only a single dose [21]. About half of cases were among those ≥ 15 years of age.

Beyond failure to vaccinate, adolescents are at increased risk for some infectious diseases because of waning immunity to vaccines administered during childhood or early adolescence [22], necessitating booster doses to prolong immunity through life. For this reason, the WHO recommends a DTaP vaccine booster at age 9 to 15 years [4]. Another example is meningococcal disease, for which recommendations are evolving. Serogroup C meningococcal (MenC) boosters for adolescents are recommended in Spain and Ireland. The MenACWY vaccination is now recommended for adolescents in Austria, Italy, and the UK (where the MenACWY program began in 2015 in response to increasing serogroup W disease rates [11]); these last countries also mandate MenC vaccination for toddlers [23].

Situations involving adolescents with poor access to healthcare (e.g., immigrants, refugees) or traveling abroad exacerbate their risk of contracting vaccine-preventable diseases. For example, an ongoing measles outbreak in Brazil is linked to the heavy influx of unvaccinated Venezuelan refugees [24].

## Reasons for low vaccine uptake among adolescents

Although immunization is firmly rooted in early childhood healthcare, the last 2 decades have witnessed the integration of routine vaccinations into adolescent healthcare [25]. However, existing recommendations are contrasted by a lack of information, education, communication, and value regarding adolescent vaccination [26–28] in public health campaigns and among HCPs, parents, and adolescents themselves. Parents’ attitudes, in particular, strongly influence the extent of vaccination coverage, and their lack of knowledge regarding adolescent vaccination recommendations can therefore impede uptake [26]. The lack of information and education among adolescents themselves was evidenced in a recent European survey in which nearly one-third of respondents reported that vaccines can be dangerous, only 60% believed that vaccines were needed at all stages of life, and only one-half were aware that meningitis is contagious [29]. Additional studies indicate that adolescents do not fully appreciate the risks associated with various vaccine-preventable diseases, such as HPV infection and meningitis [25].

Structural barriers may impede adolescent vaccination in some cases, such as the lack of easily accessible platforms to administer vaccinations during routine primary care visits [10] in some areas, or underutilization of immunization information systems [26]. Additionally, many providers have inadequate office resources (e.g., financial resources, staffing, patient-tracking systems, and vaccine storage and management) to support adolescent vaccination [26, 30]. These challenges are likely amplified by a lack of clarity on 2 fronts: ownership of adolescent vaccination decisions (by adolescents and parents) and relative responsibilities of organizations (e.g., schools, public health authorities) to provide vaccination venues.

Missed vaccination opportunities during medical care are a major concern during adolescence. Many adolescents do not have regular preventive care visits with a primary HCP [31]. It is therefore imperative to consider vaccination opportunities beyond such visits [28], including medical visits (even in emergency settings) for various concerns: sexuality, contraception orientation, and acute illness or injury; failure to do so leads to missed opportunities for adolescent vaccination [26, 32]. A visit for one vaccination can even be a missed opportunity if the other indicated vaccinations are not presented as a “package” [32]. Missed opportunities occur more frequently as adolescents age and transition from pediatricians to general practitioners and/or specialists who tend to be less focused on vaccination [26, 28, 31, 32].

Some of these barriers may be variably pronounced depending on the healthcare system of a given country and how it reaches adolescents. For example, experience suggests that Italian adolescents up to age 14 years are generally followed up by pediatricians (who regularly conduct health evaluations), whereas older adolescents are followed up by general practitioners (who do not conduct regular evaluations). In Brazil, public campaigns are held at least twice annually, and pharmacists do not have the ability to vaccinate. Notably, Brazilian vaccination calendars differ between free governmental health units (where not all vaccines are available) and the Brazilian Immunization Society [33, 34]. Where they exist, school programs aimed at teens generally result in relatively high immunization uptake; in the UK, teen-targeted school-based HPV and meningococcal vaccination programs have each reported coverage rates exceeding 80% in the first year alone, with increased HPV vaccination coverage observed in subsequent years [11, 35]. By contrast, among US adolescents, coverage with at least 1 MenACWY vaccine dose only reached 86.6% in 13 years after the initial recommendation, and up-to-date HPV vaccine coverage in 2018 was 53.7% among girls and 48.7% among boys (12 and 7 years after the initial recommendation, respectively) [13].

Adolescent vaccination introduces particular ethical challenges relating to adolescents’ autonomy, which HCPs are mandated to respect, associated with the adolescents’ desire to be involved in vaccination decisions [36]. Compared with adults, adolescents tend to have less confidence in vaccine benefits and more concern about vaccine safety [36]; adolescents also do not draw a strong connection between current actions (e.g., smoking or sexual activity) and future health [37]. These attitudes complicate the challenges of navigating and negotiating vaccination in this age group. HCPs must therefore listen carefully to the queries and concerns voiced by both adolescents and parents and provide them with accurate, comprehensive information regarding vaccination risks and benefits.

Although the Internet is a powerful tool for disseminating new health information, it carries the risk of misinformation. Adolescents may have difficulty distinguishing high- and low-quality source materials, potentially amplifying and reinforcing vaccine hesitancy [27]. Recent surveys including Australian and US adolescents emphasize the Internet’s popularity as a source of health information in this age group [38, 39]. Respondents to an Australian survey reported being most comfortable obtaining sexual health information from websites (85%), a general practitioner or doctor (81%), and school (73%) [38]. Similarly, up to 84% of surveyed US adolescents reported having searched for health information online, despite most commonly having named their parents (55%), school health classes (32%), and HCPs (29%) as sources for “a lot” of health information [39]. Notably, both groups were unlikely to use social networking sites for health information: respondents to the Australian study were least comfortable obtaining such information from various mobile device applications (51%), text messages (44%), and Twitter (36%) [38], and 88% of US teens surveyed were unlikely to seek advice or post questions on social networking sites [39]. HCPs should discuss potential sources for health information with adolescents and parents and recommend sites for reputable information.

## Strategies to improve adolescent vaccination rates

Effective adolescent vaccination strategies tend to share several key features. First, they are characterized by political will and strong leadership from a credible, well-known organization (e.g., Public Health England, Bill & Melinda Gates Foundation) that propels a given program forward. Second, they involve multiple stakeholders—adolescents, parents, educators, HCPs, public health agencies, schools and universities, public campaigns, and politicians—in developing and executing the program. Third, successful programs make benefits of vaccination clearly visible to the public through use of motivational drivers and rewards for achieving objectives. Importantly, many vaccination programs often fail to prioritize communicating success of the program to the public, with this information instead often remaining within the healthcare domain; emphasis should be placed on regularly relaying positive outcomes of vaccination programs to the public in order to further motivate vaccination. Fourth, effective programs nuance messages and methods by age group (e.g., younger vs older adolescents), recognizing that barriers may differ between groups. Finally, successful strategies focus on providing evidence-based information and education that are comprehensive but also youth-friendly, in that they create a positive emotional connection to vaccination that is attractive to, and resonates with, adolescents and their parents.

One recent excellent example of a successful adolescent vaccination program with political and public acceptance is the HPV/cervical cancer vaccination program in the UK. This UK-wide program was created to introduce school-based and primary care−based vaccination against HPV for girls aged 12 to 13 years, with catch-up vaccination available until age 18 years [35]. Significant political support for the HPV vaccination program was evidenced by the budgetary support. Vaccination was fully funded and free to recipients, and the full cost of program implementation was covered, enabling robust distribution arrangements, preparation of national and local communication materials, measurement of vaccine coverage in place, and establishment of adverse event surveillance. Targeted messaging was a central feature of the program’s rollout, evidenced by research on target groups and resource availability beginning 3 years before the program launch. Communication strategies and materials were developed based on extensive market research with multiple stakeholders, including parents, adolescents, and health professionals, and paper leaflets discussing the benefits of vaccination and containing consent forms were provided to all eligible participants. Additionally, newspaper and targeted magazine advertising were timed to coincide with the program launch, with information about the vaccination program also disseminated through television and radio advertising, public outdoor advertisements, and diverse social media platforms. This multichannel, targeted approach has been highly successful; it is estimated that > 2.3 million English girls have received the HPV vaccine series from the program’s beginning in 2008 to 2015 [35]. For the 2013/2014 vaccination cohort, coverage rates for the first, second, and third vaccine doses were 91.1%, 89.9%, and 86.7%, respectively [35].

Despite the success of this program, a similar approach cannot necessarily be easily emulated in other countries due to lack of available resources and communication among groups responsible for new initiatives targeting adolescent and young adult vaccination, such as governmental agencies and health professional associations. For example, as mentioned previously, HPV vaccine coverage in the USA in 2018 was approximately 50% among adolescent girls and boys [13]. Efforts to increase vaccine coverage have been hindered by several factors including provider practices, lack of information for parents and physicians, financial barriers (e.g., insufficient insurance coverage), safety concerns, and parental attitudes (e.g., vaccine misconception and association with sexual activity) [40].

## A call to action

HCPs play a key role in encouraging and facilitating any opportunity for adolescent vaccination, with the strength of a provider’s recommendation alone (e.g., the difference between “I recommend you receive these vaccines today” and “What would you like to do about today’s vaccinations?”) being one of the most important drivers [41]. Multiple studies have identified HCPs as those whom parents trust most for vaccine safety information and recommendations for adolescent vaccination [26].

Building trust with adolescents and parents requires ongoing work, with measured responses to events or situations that may erode that trust [42]. HCPs should take advantage of every visit to address vaccination with adolescents, regardless of the reason for the visit. They should also remain educated and updated on vaccine-preventable diseases and vaccination recommendations for adolescents, and take time to understand the perspectives of adolescents and their parents when discussing the benefits and risks of vaccination. Evidence suggests that parents are willing to learn more and discuss vaccinations if given appropriate opportunities and resources [43, 44]. A UK study found that more than half of adolescents prefer discussing vaccination decisions with their parents, which underscores the need for parents to be well-informed [45]. Thus, educational campaigns through various channels (e.g., media, advertisements), with targeted messaging to reach both parents and adolescents in the general public is of great importance.

Because successful vaccination strategies rely on coordinated action between multiple stakeholders, it is incumbent upon public health authorities to increase availability of evidence-based information about vaccines and the diseases they target as well as vaccination campaigns. The ultimate goal should be communicating successes achieved through vaccination programs, thereby counteracting, in real time, doubts regarding vaccine safety and efficacy that ultimately contribute to vaccine hesitancy. Consistent, lasting efforts should be made to use multiple media streams, including outdoor advertising, radio, television, video, and Internet social media networks to disseminate youth-friendly messages that present vaccination as part of a positive and healthy adult lifestyle. Furthermore, adolescent immunization should be undertaken as a local, regional, national, and global cause, with appropriate leaders appointed at each level. Finally, health authorities should continue to focus on successful vaccination strategies targeting adolescents, implement these strategies to successfully improve adolescent vaccination rates, and make these results transparently available to all stakeholders, particularly adolescents and the public.

## Conclusions

Although adolescence is often marked by an increase in risky behavior that can favor certain vaccine-preventable diseases, vaccination rates in this age group remain low, especially when compared with those in infants and toddlers. Specific barriers at the levels of the health system, HCPs, parents, and adolescents impede adolescent vaccination, particularly regarding lack of education and information on immunization. Thus, the success of vaccination programs in this age group especially requires coordinated and unified action between these different stakeholders. Success also relies on the crucial role of public health agencies to implement widespread vaccination strategies; these agencies should therefore mobilize to champion this important cause at every regional level. At the point of care, HCPs are key players in parents’ and adolescents’ decision to vaccinate; they must remain abreast of adolescent vaccination recommendations and prioritize adolescent vaccination. Raising awareness among parents and adolescents through effective communication regarding the benefits and risks of vaccination is critical to improvement.

## Abbreviations

DTaP: Diphtheria, tetanus, and acellular pertussis
HCP: Healthcare provider
HPV: Human papillomavirus
MenACWY: Meningococcal serogroups A, C, W, and Y
MenC: Meningococcal serogroup C
WHO: World Health Organization
